# PEEP application during mechanical ventilation contributes to fibrosis in the diaphragm

**DOI:** 10.1186/s12931-023-02356-y

**Published:** 2023-02-13

**Authors:** Xiaoli Qian, Ye Jiang, Jianwei Jia, Weimin Shen, Yuejia Ding, Yuhan He, Peifeng Xu, Qing Pan, Ying Xu, Huiqing Ge

**Affiliations:** 1 Department of Respiratory Care, Regional Medical Center for National Institute of Respiratory Diseases, Sir Run Run Shaw Hospital, School of Medicine, Zhejiang University, Qingchun East Rd. 3, Hangzhou, 310016 China; 2 College of Information Engineering, Zhejiang University of Technology, Liuhe Rd. 288, Hangzhou, 310023 China

**Keywords:** Mechanical ventilation, Diaphragm dysfunction, Fibrosis, Collagen, TGF-β1

## Abstract

**Background:**

Positive end-expiratory airway pressure (PEEP) is a potent component of management for patients receiving mechanical ventilation (MV). However, PEEP may cause the development of diaphragm remodeling, making it difficult for patients to be weaned from MV. The current study aimed to explore the role of PEEP in VIDD.

**Methods:**

Eighteen adult male New Zealand rabbits were divided into three groups at random: nonventilated animals (the CON group), animals with volume-assist/control mode without/ with PEEP 8 cmH_2_O (the MV group/ the MV + PEEP group) for 48 h with mechanical ventilation. Ventilator parameters and diaphragm were collected during the experiment for further analysis.

**Results:**

There was no difference among the three groups in arterial blood gas and the diaphragmatic excursion during the experiment. The tidal volume, respiratory rate and minute ventilation were similar in MV + PEEP group and MV group. Airway peak pressure in MV + PEEP group was significantly higher than that in MV group (*p* < 0.001), and mechanical power was significantly higher (*p* < 0.001). RNA-seq showed that genes associated with fibrosis were enriched in the MV + PEEP group. This results were further confirmed on mRNA expression. As shown by Masson’s trichrome staining, there was more collagen fiber in the MV + PEEP group than that in the MV group (*p* = 0.001). Sirius red staining showed more positive staining of total collagen fibers and type I/III fibers in the MV + PEEP group (*p* = 0.001; *p* = 0.001). The western blot results also showed upregulation of collagen types 1A1, III, 6A1 and 6A2 in the MV + PEEP group compared to the MV group (*p* < 0.001, all). Moreover, the positive immunofluorescence of COL III in the MV + PEEP group was more intense (*p* = 0.003). Furthermore, the expression of TGF-β1, one of the most potent fibrogenic factors, was upregulated at both the mRNA and protein levels in the MV + PEEP group (mRNA:* p* = 0.03; protein:* p* = 0.04).

**Conclusions:**

We demonstrated that PEEP application for 48 h in mechanically ventilated rabbits will cause collagen deposition and fibrosis in the diaphragm. Moreover, activation of the TGF-β1 signaling pathway and myofibroblast differentiation may be the potential mechanism of this diaphragmatic fibrosis. These findings might provide novel therapeutic targets for PEEP application-induced diaphragm dysfunction.

**Supplementary Information:**

The online version contains supplementary material available at 10.1186/s12931-023-02356-y.

## Background

Positive end-expiratory airway pressure (PEEP) is a potent component of management for patients receiving mechanical ventilation (MV). For critically ill patients, PEEP improves gas exchange, increase end expiratory lung volume (EELV) and improves pulmonary homogeneity, improves clinical outcomes including mortality [1, 2]. However, recently, evidence has shown that the prolonged application of PEEP during mechanical ventilation may cause diaphragm remodeling, especially longitudinal muscle fiber atrophy [3]. PEEP may lead to the development of ventilation-induced diaphragm dysfunction (VIDD), making it difficult for patients to be weaned from MV.

Early studies had a particular focus on the effect of ventilator assistance (assisted or controlled ventilation) on diaphragm dysfunction. However, little attention has been given to the role of PEEP in this process. A variety of studies have shown that lower tidal volume (V_T_) and higher PEEP can play a lung protective role in mechanical ventilation [4–7]. However, the increased end-expiratory lung volume (EELV) caused by high PEEP affect diaphragm geometry. Thus, the diaphragm is always subjected to mechanical forces from the lungs, which may be related to diaphragmatic dysfunction due to mechanical ventilation [3]. Recently, Lindqvist found that ventilation with PEEP resulted in diaphragm remodeling [8]. Thus, further understanding of the potential mechanism in PEEP application-induced diaphragm dysfunction is crucial for establishing strategies to guide clinical practice.

Repair normally occurs very quickly after tissue undergoes mechanical trauma, exposure to toxins or infections. However, successful repair involves a series of complicated and well-orchestrated events; otherwise, failed tissue repair, including degeneration, inflammation, and fibrosis, will occur. Fibrosis is the aberrant or dysregulated accumulation of extracellular matrix (ECM) components, especially collagens, in the process of tissue repair, leading to organ/tissue dysfunction [9, 10]. Fibrosis causes muscle dysfunction. With the accumulation of fibrotic tissue, muscle stiffness increases and obstructs the diaphragm to reach the offset length required for respiration. In addition, a study found that changes in collagen organization structure and mechanics have an impact on diaphragm function [11]. In skeletal muscle, transforming growth factor-β1 (TGF-β1) is considered one of the most effective regulators in the process of fibrosis, as it controls ECM synthesis, remodeling, and degradation [12]. The hyperactivation of TGF-β1 eventually contributes to the conversion of muscle fiber into nonfunctional fibrotic tissue and the dysfunction of force generating capacity in skeletal muscle [12, 13]. Hence, targeting fibrosis may be one of the therapeutic strategies in mechanical ventilation with PEEP application.

In this context, we explored whether high PEEP application increases collagen deposition and promotes fibrosis in the diaphragms of rabbits with MV, which may provide new insights in pathophysiology and treatment of VIDD.

## Methods

### Study design

The purpose of this study was to evaluate the effect of the application of PEEP on the diaphragm during mechanical ventilation. This treatment was studied in a rabbit mechanical ventilation model.

Eighteen adult male New Zealand rabbits weighing 2.5 ± 0.2 kg were included in this study. The rabbits were randomly divided into three groups (six per group) based on a list generated by a random number generator in Excel. Group 1: nonventilated animals under sedation (CON group); Group 2: animals with mechanical ventilation with a PEEP of 0 cmH_2_O (MV group); Group 3: animals with mechanical ventilation with a PEEP of 8 cmH_2_O (MV + PEEP group). All animals were euthanized after 48 h of ventilation, arterial blood gas (pH value, arterial partial pressure of carbon dioxide and arterial partial pressure of oxygen, etc.) was measured, and diaphragm muscle samples were collected for subsequent analyses.

### Animals

**Fig. 1:**
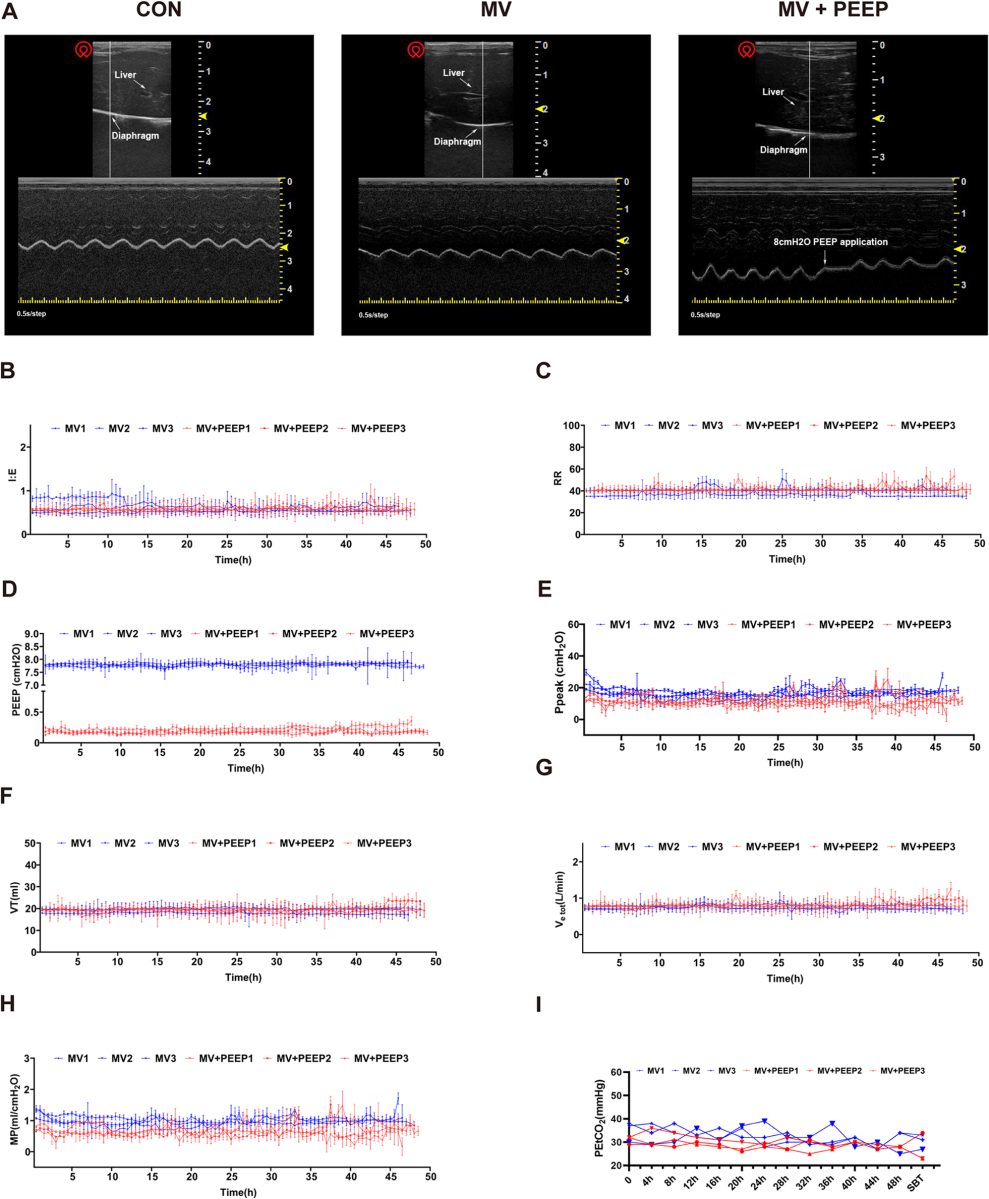
Diaphragm ultrasonic and respiratory parameter monitoring during model establishment. **A** Diaphragm ultrasonic analysis revealed the frequency of motion and the location of the diaphragm. **B** Respiratory parameter monitoring of I:E. **C** Respiratory parameter monitoring of RR (cpm). **D** Respiratory parameter monitoring of PEEP (cmH_2_O). **E** Respiratory parameter monitoring of Ppeak (cmH_2_O). **F** Respiratory parameter monitoring of VT (ml). **G** Respiratory parameter monitoring of Ve tot (**L**). **H** Respiratory parameter monitoring of MP(J/min). **I** Respiratory parameter monitoring of PETCO_2_ (mmHg). Three rabbits from each group were randomly selected to draw the trend line of respiratory parameter

MV group and MV + PEEP group animals were mechanically ventilated, and anesthesia was performed by intraperitoneal injection of 3% sodium pentobarbital solution (1 ml/kg) and injecting lidocaine in the neck at the incision site to reduce pain. Moreover, a venous indwelling needle was established under the vein of the rabbit ear. Anesthesia was maintained with an intravenous pentobarbital sodium infusion. A feeding tube was inserted into the stomach via a small incision in the esophagus. The ventilator used in the experiment was the SV800 neonatal circuit ventilator (neonatal type: Mindray, Shenzhen, China). In the experiment, the volume assist/control mode was used, the tidal volume was 8 ml/kg, the respiratory rate was 40–50 bpm, and the PEEP level was 0 cm H_2_O or 8 cm H_2_O. As shown in Fig. 1, the end-tidal CO_2_ pressure (PETCO_2_) was monitored every 4 h during the experiment, and the ventilator parameters were adjusted according to the values. The pressure and airflow waveforms of the ventilator were continuously monitored through the RS-232 output port of the ventilator, and the respiratory parameters were continuously recorded similarly (RespcareRM, Hangzhou ZhiRuiSi Company, China). The spontaneous breathing trial (SBT) was performed after 48 h of mechanical ventilation. The ventilator mode was changed to spontaneous breathing mode (both the pressure support and PEEP used were 0 cm H_2_O). During this period, parameters such as respiratory rate (RR), tidal volume (V_T_), total minute ventilation (V_e tot_), and rapid shallow breathing index (RSBI) were collected. Diaphragmatic ultrasound assessments were performed before the experiment, at 48 h of mechanical ventilation, and during SBT. All animal experimental protocols were approved by the Review Committee of Zhejiang University School of Medicine.

### Histological analysis

Harvested diaphragm muscles were processed to the appropriate size, immediately fixed in 4% paraformaldehyde and processed for paraffin embedding. Then, 8 μm thick sections were prepared. The diaphragm tissue was processed by Masson's trichrome staining and picric acid-Sirius red staining. The diaphragm specimen was stained with 0.1% Sirius red in a saturated aqueous solution of picric acid for 50 min at 37 °C. Then, the tissue sections were washed in 0.01 N HCl to remove unbound dye, dehydrated with 100% ethanol, and washed in xylene. Masson's trichrome staining was performed following the Trichrome Staining Kit manual. The diaphragm slices were observed under a microscope (Olympus VS200, Tokyo, Japan). Specifically, the sections with picric acid-Sirius red staining were observed under polarized light. The percentage of collagen in diaphragm sections was measured with Image-Pro-Plus software, and five separate views were selected.

### Immunofluorescence

Six-micrometer-thick paraformaldehyde-fixed, paraffin-embedded sections of diaphragm were deparaffinized for immunofluorescent staining. All sections were incubated with anti-collagen III primary antibody (HA720050 1:500 HuaAn Biotechnology, China) at 4 °C overnight. Tissue was then incubated with secondary antibodies against rabbit (A-11035, 1:500, Invitrogen, United States) labeled with Alexa Fluor 546 at 37 °C for 30 min. Then, all slides were stained with Hoechst (H1399, 1:500, Invitrogen, United States) and wheat germ agglutinin (WGA) (W11261, 1:1000, Invitrogen, United States) for 10 min 37 °C. Finally, the slides were observed under a confocal microscope (Olympus IX83-FV3000-OSR, Tokyo, Japan), and representative views were selected and photographed.

### Quantitative RT‒qPCR

Total RNA was extracted using an AxyPrep Multisource Total RNA Maxiprep Kit (AP-MX-MS-RNA-10G, Axygen, United States). cDNAs were synthesized using the HiScript® II Reverse Transcriptase Kit (R223, Vazyme, China). qPCR was performed following the manufacturer’s protocol (Q711, Vazyme, China). The primers used in this study are listed in Additional file 1: Table S1. Target genes included COL1A1, COL1A2, COL3A1, COL5A3, COL6A1, COL6A2, COL15A1, COL16A1, TGF-β1, FN, THBS1 and THBS3. GAPDH was adopted as the housekeeping gene, and the relative expression values of target genes were calculated using the 2^−ΔΔCt^ method.

### Western blot analysis

The diaphragm protein lysates were subjected to SDS‒PAGE gels for electrophoresis for 90 min and then transferred onto a polyvinylidene fluoride membrane (IPVH00010, Millipore, United States). The membrane was blocked and then incubated with primary antibodies against TGF-β1 (HA500496 1:1000, HuaAn Biotechnology, China), α-SMA (ER1003 1:1000, HuaAn Biotechnology, China), COL3 (HA720050 1:1000, HuaAn Biotechnology, China), COL1A1 (ET1609-68, 1:1000, HuaAn Biotechnology, China), COL6A1 (A9236, 1:1000, ABclonal, Woburn, MA, USA), and COL6A2 (A3796, 1:1000, ABclonal, Woburn, MA, USA) overnight at 4 ℃ and secondary antibodies at room temperature for 90 min and then incubated with ECL reagent. The final reported data of COL1A1, COL3, COL6A1, COL6A2, TGF-β1 and α-SMA were the band densities normalized to that of GAPDH.

### RNA-seq analysis

RNA-seq was performed with 2 × 150 bp paired-end sequencing (PE150) on an Illumina Novaseq™ 6000 (LC-Bio Technology Co., Ltd., Hangzhou, China) following the protocol. Then, analysis of significant differences, GO enrichment and KEGG enrichment analyses were performed on the differentially expressed mRNAs.

### Statistical analysis

Comparisons between of three groups were conducted by one-way analysis of variance (ANOVA) and nonparametric tests, and the values are presented as the means ± SDs. Statistical significance was considered when the *p* value was less than 0.05 by SPSS 19.0 statistical software.

## Results

### Physiological measurements and respiratory monitoring during model establishment

The initial body weights of the CON group, the MV group and the MV + PEEP group were 2.52 ± 0.09 kg, 2.56 ± 0.05 kg and 2.43 ± 0.02 kg, respectively. The respiratory parameters were monitored throughout (Fig. 1B–H and Additional file 1: Table S2), which indicated that the respiratory strategy we carried out provided sufficient ventilation support and maintained a good breathing pattern for rabbits. Moreover, the airway peak pressure (Ppeak) and mechanical power (MP) in the MV group were significantly lower than that in the MV + PEEP group (Ppeak: 11.52 ± 1.24 cmH2O and 15.88 ± 0.85 cmH2O, respectively, *p* < 0.001 MP: 0.66 ± 0.09 J/min and 0.99 ± 0.05 J/min, respectively, *p* < 0.001).

As shown in Table 1, none of the blood gas parameters at the end of the experiment were significantly different among groups. We found that the results of SBT between the MV group and the MV + PEEP group were not significantly different after mechanical ventilation (Table 2). After spontaneous breathing trial, the parameters of PETCO_2_ between the MV group and the MV + PEEP group were 29.50 ± 7.74 mmHg and 31.33 ± 4.59 mmHg, respectively.

**Table 1:** Body weight and blood gas analysis in three groups

	CON	MV	MV + PEEP	^*$*^*p*
Body weight (kg)	2.52 ± 0.09	2.56 ± 0.05	2.43 ± 0.02	0.076
Lac (mmol/L)	3.08 ± 0.63	3.15 ± 2.89	3.63 ± 3.57	0.810
PH	7.35 ± 0.03	7.39 ± 0.07	7.34 ± 0.13	0.440
HCO_3_^−^ (mmol/L)	22.20 ± 2.00	20.05 ± 1.48	20.37 ± 4.1	0.875
PaCO_2_ (mmHg)	40.15 ± 5.93	34.2 ± 4.74	38.43 ± 10.17	0.444
PaO_2_ (mmHg)	57.98 ± 25.45	59.70 ± 26.45	58.83 ± 15.71	0.963
FiO2(%)	21.00	21.00	21.00	
PaO_2_/FiO_2_ (mmHg)	276.07 ± 121.20	284.26 ± 125.93	280.16 ± 74.81	0.963
SBE,c	− 3.35 ± 1.63	− 4.63 ± 2.60	− 5.47 ± 5.70	0.755
PETCO_2_ (mmHg)	NA	29.50 ± 7.74	31.33 ± 4.59	0.629

Data are expressed as mean ± SD

^$^*p* MV vs. MV + PEEP

**Table 2:** SBT parameters in MV group and MV + PEEP group

	CON	MV	MV + PEEP	*p*
RR ( cpm)	NA	62.97 ± 19.62	55.52 ± 12.03	0.546
RSBI ( cpm /L)	NA	4330.91 ± 1805.15	4465.98 ± 2389.68	0.447
V_e tot_ (L)	NA	0.80 ± 0.12	0.92 ± 0.32	0.401
VT (ml)	NA	16.67 ± 3.95	18.91 ± 5.81	0.453

Data are expressed as mean ± SD

Diaphragm ultrasound can reveal the frequency of motion and the location of the diaphragm (Fig. 1A). When we applied 8 cm H_2_O PEEP to the diaphragm, the location of the diaphragm was altered and maintained in the new position under the persistent mechanical forces of PEEP. We detected the diaphragm excursion of these three groups before mechanical ventilation, after 48 h of mechanical ventilation and after SBT, as shown in Table 3. We found that mechanical ventilation with or without PEEP application had little influence on diaphragm excursion.

**Table 3:** Diaphragm excursion measured by ultrasound

	CON	MV	MV + PEEP
Before mechanical ventilation(mm)	3.97 ± 1.20	3.81 ± 1.17	3.91 ± 0.60
During mechanical ventilation(mm)	NA	3.80 ± 0.70	3.77 ± 0.55
SBT(mm)	3.85 ± 0.51	3.88 ± 1.21	3.63 ± 1.19

Data are expressed as mean ± SD

### PEEP application during mechanical ventilation leads to extracellular matrix alteration and collagen deposition

**Fig. 2:**
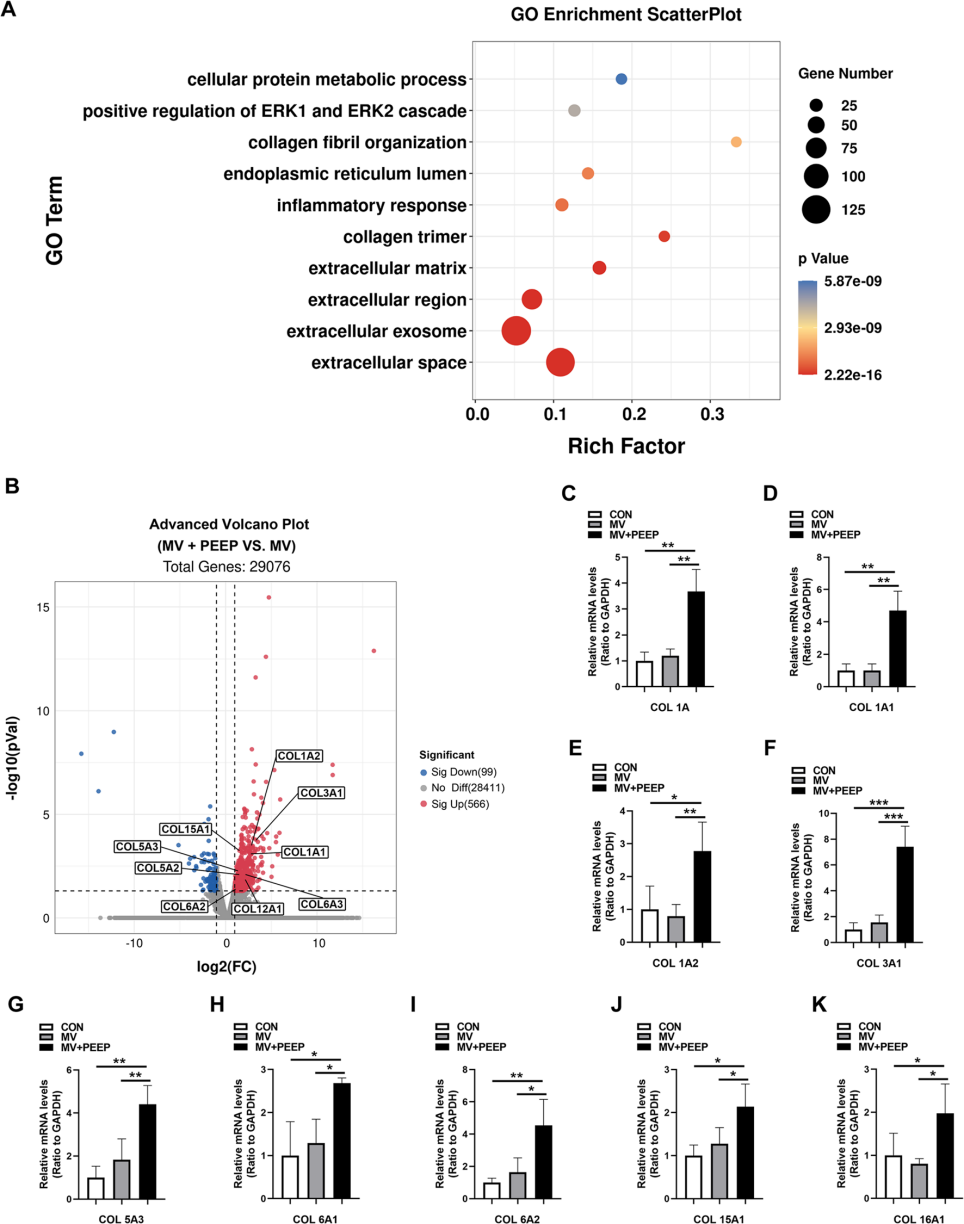
Fibrosis participates in ventilation-induced diaphragm dysfunction. **A** Bubble chart of the top 10 categories for GO enrichment of the MV group and the PEEP group (n = 3). **B** Volcano plot of differential gene expression of the MV group and the PEEP group (n = 3). **C**–**K** The diaphragm of the two groups was subjected to qPCR to analyze different type of collagen expression. Values are represented as the mean ± SD (*p < 0.05, **p < 0.01, ***p < 0.001)

According to the RNA-seq results, we identified a total of 665 differentially regulated genes among 29,076 genes between the MV group and the MV + PEEP group. Among these, the levels of 566 genes were significantly upregulated, and 99 were significantly downregulated (Fig. 2B). Interestingly, Gene Ontology (GO) analysis of differentially expressed genes showed that among the top 10 significantly enriched GO terms, 4 GO terms were associated with extracellular matrix and fibrosis (Fig. 2A). This finding indicated that the application of PEEP during mechanical ventilation may alter the extracellular matrix of the diaphragm. By further probing our data, we found some differentially regulated genes associated with collagen, as shown in Fig. 2B. Moreover, the mRNA expression levels of these significantly regulated genes were further verified to be increased in the MV + PEEP group compared to the MV group (COL 1A: 1.20 ± 0.26 and 3.68 ± 0.85, respectively, *p* = 0.002; COL 1A1: 1.00 ± 0.41 and 4.70 ± 1.20, respectively, *p* = 0.001; COL 1A2: 0.79 ± 0.36 and 2.78 ± 0.86, respectively, *p* = 0.008; COL 3A1: 1.56 ± 0.57 and 7.414 ± 1.60, respectively, *p* = 0.001; COL 5A3: 1.83 ± 0.97 and 4.40 ± 0.88, respectively, *p* = 0.008; COL 6A1: 1.29 ± 0.56 and 2.68 ± 0.12, respectively, *p* = 0.023; COL 6A2: 1.64 ± 0.86 and 4.53 ± 1.61, respectively, *p* = 0.014; COL 15A1: 1.28 ± 0.38 and 2.14 ± 0.53, respectively, *p* = 0.032; COL 16A1: 0.81 ± 0.12 and 2.44 ± 1.09, respectively, *p* = 0.026) (Fig. 2C–K). These results indicated that the application of PEEP during mechanical ventilation may lead to extracellular matrix alterations and collagen deposition.

### PEEP application during mechanical ventilation contributes to diaphragm fibrosis

**Fig. 3:**
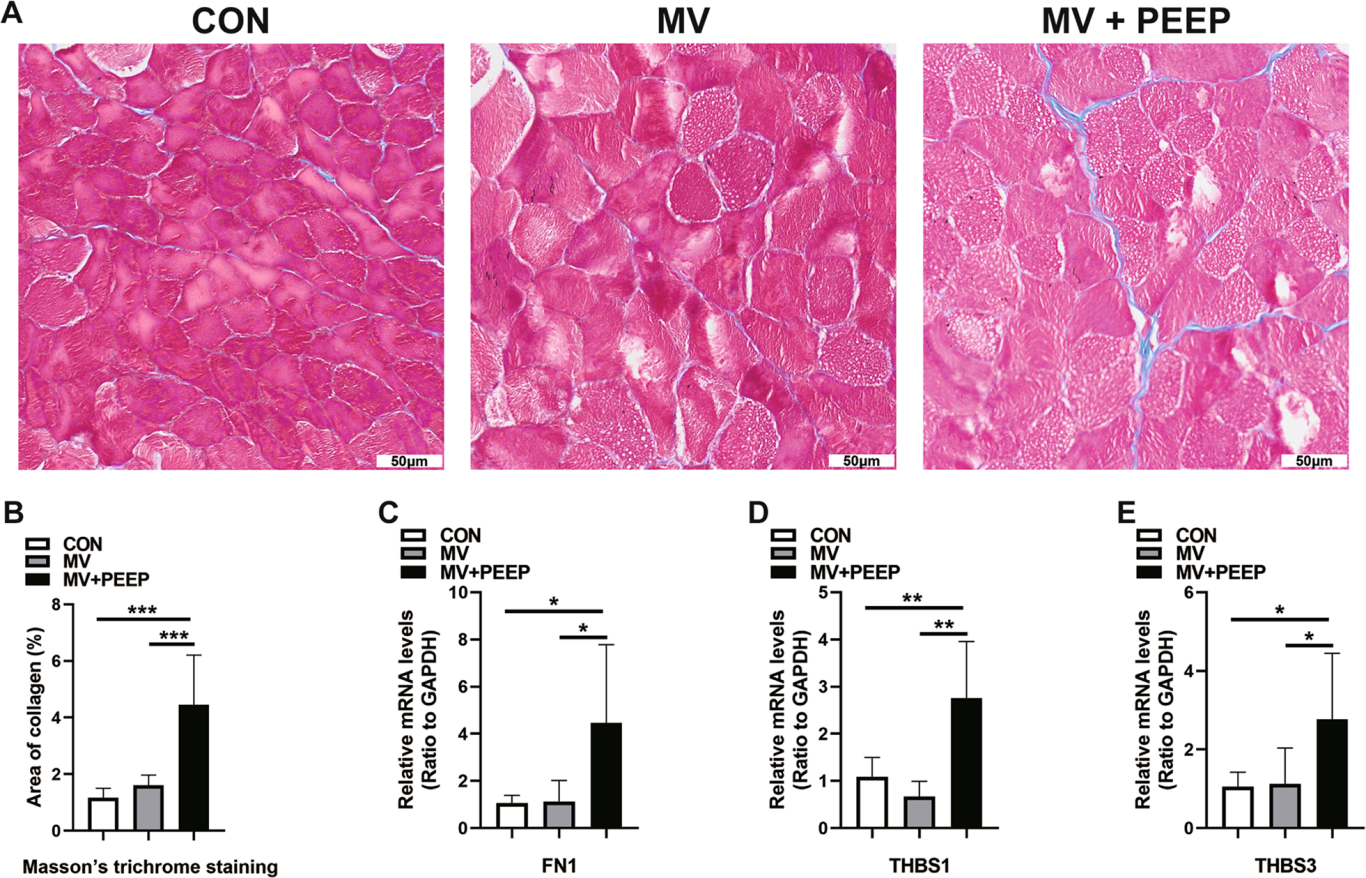
Application of PEEP during mechanical ventilation leads to diaphragm fibrosis. **A**, **B** Masson’s trichrome staining revealed fibrosis in the diaphragms of the MV + PEEP group. Scale bar = 50 μm. **C**–**E** Diaphragms of three groups were detected by qPCR to analyze fibrosis-related gene expression. Values are represented as the mean ± SD, n = 6 (*p < 0.05, **p < 0.01, ***p < 0.001)

To further investigate the influence of extracellular matrix alteration and collagen deposition on the diaphragm, we carried out Masson’s trichrome staining to identify collagen fiber accumulation. Masson’s trichrome can stain collagen fibers of the diaphragm blue and muscle fibers red. As shown in Fig. 3A and B, the collagen fiber staining in the MV + PEEP group was stronger than that in the MV group (1.60 ± 0.37 and 4.45 ± 1.76, respectively, *p* = 0.001). Additionally, we found that other extracellular matrix and collagen-related mRNAs were upregulated in the MV + PEEP group compared to the MV group (FN: 1.13 ± 0.89 and 4.49 ± 3.28, respectively, *p* = 0.05; THBS1: 0.67 ± 0.32 and 2.76 ± 1.20, respectively, *p* = 0.006; THBS3: 1.12 ± 0.92 and 2.76 ± 1.68, respectively, *p* = 0.05) (Fig. 3C–E).

**Fig. 4:**
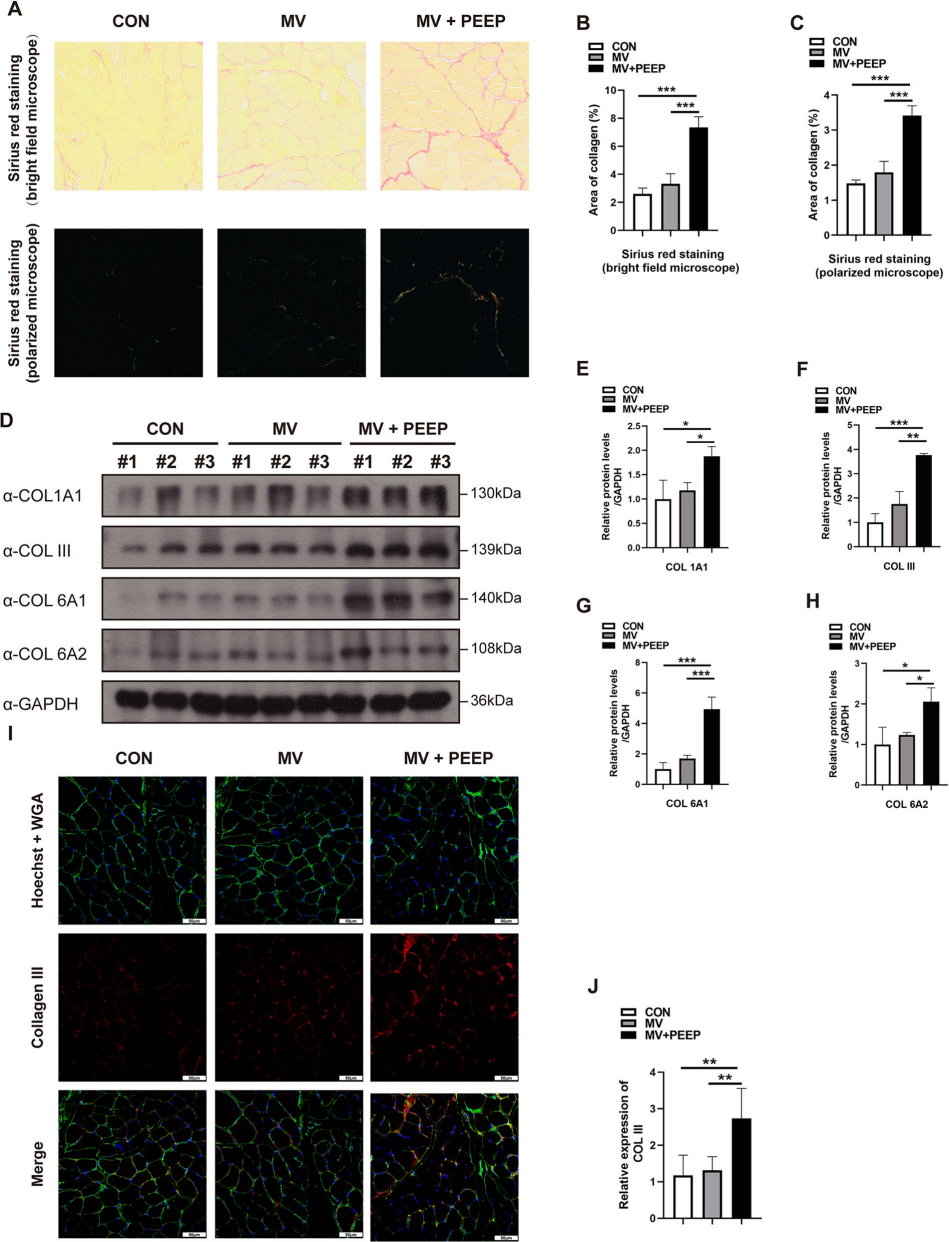
Application of PEEP during mechanical ventilation leads to collagen deposition in the diaphragm. **A**–**C** Sirius red staining with bright field and polarized microscopy revealed increased collagen fibers in the diaphragms of the MV + PEEP group. Scale bar = 50 μm. **D**–**H** Diaphragm protein lysates of the three groups were detected by Western blotting, and the expression of different collagen types was quantified. **I**, **J** Diaphragmatic tissue sections were subjected to immunofluorescence staining to analyze the expression of collagen III. Red represents collagen III; green represents WGA; blue represents Hoechst. Scale bar = 50 μm. Values are represented as the mean ± SD. n = 6 (*p < 0.05, **p < 0.01, ***p < 0.001)

We next investigated the alteration of different collagen types. Sirius red staining is another standard method for evaluating collagen fibers in tissues. The complex of collagen and Sirius red is much more birefringent than that of other proteins; thus, it appears brighter than the other tissues with polarized microscopy [14]. Moreover, collagen type I fibers can appear yellowish-orange to red, while collagen type III fibers appear green to yellowish-green with polarized microscopy [15]. We found that regardless of the total collagen fibers detected by the bright field microscope or the collagen type I and III fibers visualized by the polarized microscope, the MV + PEEP group presented more positive staining than the MV group (bright field microscope: 3.33 ± 0.72 and 7.35 ± 0.75, respectively,* p* = 0.001; polarized microscope: 1.80 ± 0.32 and 3.42 ± 0.28, respectively,* p* = 0.001) (Fig. 4A–C). Similar results were found by western blotting: the protein expression levels of collagen types 1A1, III, 6A1 and 6A2 were significantly increased in the MV + PEEP group compared to the MV group (COL 1A1: 1.18 ± 0.16 and 1.88 ± 0.20, respectively, *p* = 0.05; COL III: 1.75 ± 0.52 and 3.76 ± 0.07, respectively, *p* = 0.001; COL 6A1: 1.70 ± 0.21 and 4.93 ± 0.80, respectively, *p* = 0.001; COL 6A2: 1.24 ± 0.06 and 2.06 ± 0.34, respectively, *p* = 0.04) (Fig. 4D–H). We also found that compared to that of the MV group, the positive immunofluorescence of COL III in the MV + PEEP group was stronger (1.13 ± 0.37 and 2.73 ± 0.83, respectively,* p* = 0.003) (Fig. 4I, J). Taken together, our present study found that the application of PEEP during mechanical ventilation contributes to collagen fiber accumulation, indicating fibrosis in the diaphragm.

### PEEP application during mechanical ventilation induced fibrosis in the diaphragm is associating with TGFβ-1 upregulation

**Fig. 5:**
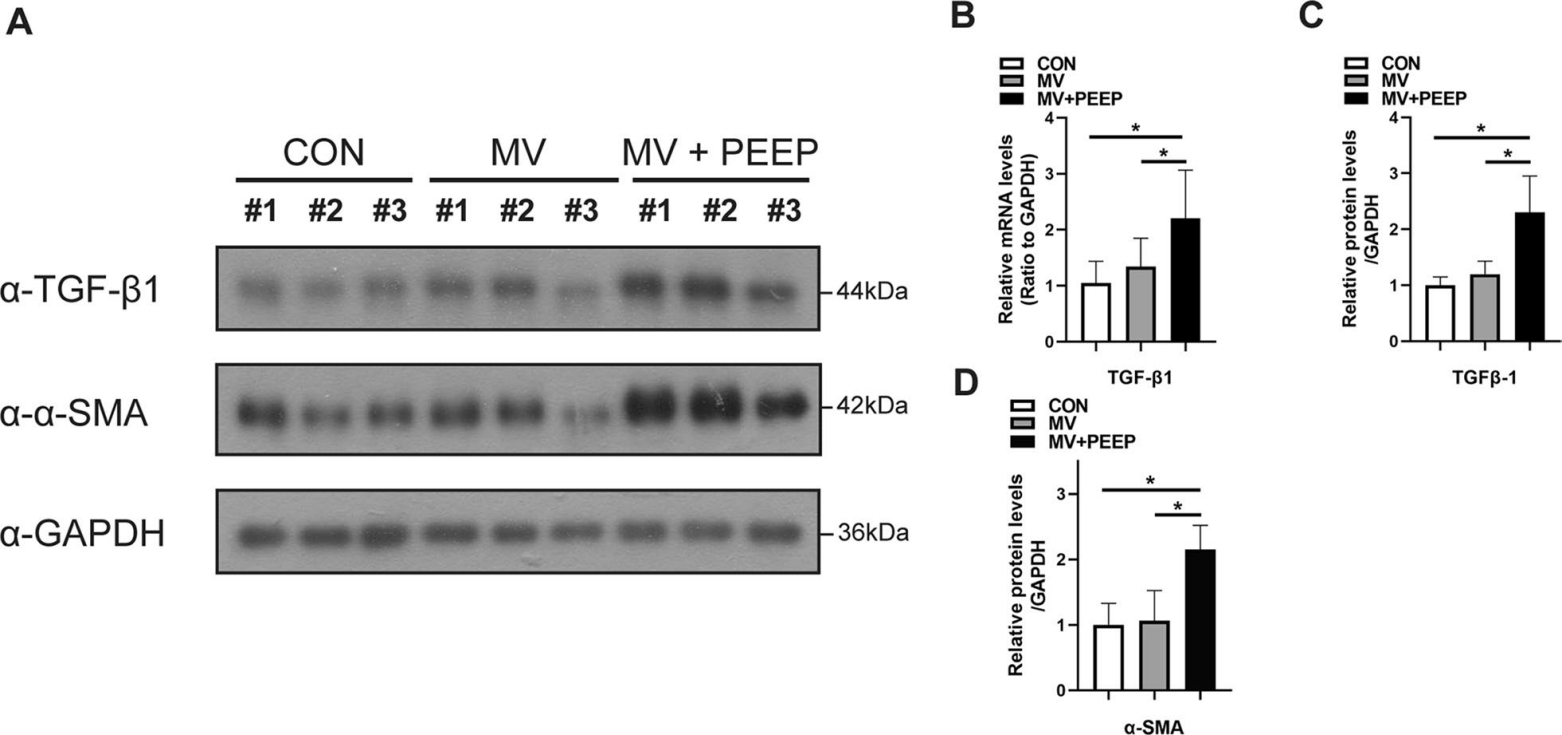
PEEP application during mechanical ventilation induced fibrosis in the diaphragm is associating with TGFβ-1 and α-SMA upregulation. **A**–**D** Diaphragm protein lysates of the three groups were detected by Western blotting, and the expression of TGFβ-1 and α-SMA were quantified. Diaphragms of the three groups were detected by qPCR to analyze fibrosis-related gene expression. Values are represented as the mean ± SD. n = 6 (*p < 0.05)

TGFβ-1 is known as one of the most effective fibrogenic factors, playing an important role in the expression of collagen fibers. We found that the expression of TGFβ-1 at both the mRNA and protein levels was upregulated in the MV + PEEP group (mRNA: 1.35 ± 0.50 and 2.42 ± 0.77, respectively,* p* = 0.03; protein: 1.20 ± 0.23 and 2.30 ± 0.65, respectively,* p* = 0.04) (Fig. 5A–C). Additionally, we found that the expression of alpha smooth muscle actin (α-SMA) in the MV + PEEP group was significantly upregulated (1.06 ± 0.46 and 2.15 ± 0.37, respectively,* p* = 0.03) (Fig. 5A, D). In summary, the upregulation of TGFβ-1 in mechanical ventilation with PEEP application may be the potential mechanism of fibrosis in the MV + PEEP group.

## Discussion

PEEP is a common and important method in acute hypoxic respiratory failure. Most acute respiratory distress syndrome (ARDS) patients who receive mechanical ventilation will receive PEEP of 5 to 12 cm H_2_O in combination with lung-protective ventilation modes to ameliorate oxygenation and prevent atelectasis [16, 17]. However, it has been demonstrated that after 18 h of MV with 2.5 cm H_2_O of PEEP, diaphragm fibers adapt to the altered shape by absorbing serially linked sarcomeres, which is termed longitudinal atrophy [8]. Recent research proved that MV with 10 cm H_2_O of PEEP for 12 h worsens diaphragm atrophy induced by a ventilator in rats by inducing oxidative stress [7]. However, the influence of PEEP application is still controversial. Sassoon et al. found that 48 h of MV with 8 cm H_2_O of PEEP did not exacerbate diaphragm dysfunction in rabbits [18]. The potential explanation is that the profound effects of CMV on the diaphragm made the additional influence of PEEP undetectable. It has even been reported that high levels of PEEP application preserved diaphragm contractility in a rat ARDS model.

In our study, we adopted PEEP of 8 cm H_2_O which has been confirmed that 8 cm H_2_O PEEP will not cause pulmonary overdistension or circulatory dysfunction [18]; In addition, the ventilation mode of volume assist/control preserves the spontaneous breathing effort of the experimental rabbits, which is closer to clinical application and protects the diaphragm function to a certain extent [19]. However, this may contribute to the patient-ventilator asynchrony which may also have impact on diaphragm. We made attempts to explore the role of patient-ventilator asynchrony on diaphragm dysfunction as shown in Additional file 1: Table S3. Based on current data, patient-ventilator asynchrony index between the MV group and the MV + PEEP group is not significant different. Interestingly, we found that mechanical ventilation with 8 cm H_2_O PEEP application led to fibrosis in the diaphragm in healthy rabbits by upregulating the expression of TGF-β1, which may be a potential cause of aggravating diaphragm dysfunction.

Fibrosis refers to the accumulation of ECM. Although ECM accounts for 10% of skeletal muscle mass and plays a major role in force transmission, maintenance, and muscle fiber repair [20], the abnormal accumulation of ECM, especially collagens, impairs muscle function and regeneration after injury [12, 21, 22]. Brass et al. demonstrated that high-fat diet (HFD) feeding promotes the role of thrombospondin 1 (THBS1) in obesity-related respiratory dysfunction by increasing FAP-mediated fibrogenesis and promoting fibrotic remodeling of the diaphragm [23]. A study of Duchenne muscular dystrophy (DMD) identified changes in the ECM structure and mechanics of the diaphragm during disease progression, and the role of collagen tissue in diaphragm function should be further investigated [11]. A previous study showed that a diaphragmatic injury model induced by high tidal volume ventilation in mice results in diaphragm dysfunction, which is associated with the activation of fibrosis-relevant proteins such as type I and III procollagen and TGF-β1. Mechanical stretch was considered to be the reason for this high tidal volume ventilation-induced fibrosis in the diaphragm [24]. Similarly, in our study, we found that PEEP application led to collagen deposition and fibrosis in a mechanical ventilated diaphragm. This finding may be due to the altered location of the diaphragm that we detected with diaphragm ultrasound (Fig. 1A). During the process of 48 h of mechanical ventilation, the diaphragm suffered an 8 cm H_2_O mechanical force. Persistent mechanical stretching might account for the fibrotic activation observed in our present research.

As an important regulator of ECM accumulation, TGF-β1 is considered to be a key driver in the development of fibrosis [25]. Several studies have demonstrated that TGF- β1 signaling is critical in the progression of several diseases, such as pulmonary fibrosis, cardiac fibrosis and liver fibrosis [26–28]. In the diaphragm of an MDX mouse model, TGF-β1 levels were significantly upregulated, accompanied by an increase in nonmuscle tissue [29]. Baptiste et al. found that a TGF-β pathway inhibitor alleviates diaphragmatic contraction dysfunction induced by sepsis [30]. In addition, TGF-β, especially TGF-β1, and its downstream signaling pathway are potent regulators of α-SMA gene expression during damage repair [31, 32]. Cumulative α-SMA expression is considered to be a classic hallmarks of myofibroblast differentiation, representing mature myofibroblasts [33]. Persistent activation of myofibroblasts leads to accumulation and contraction of collagenous ECM and eventually contributes to the development of fibrosis. In our present study, we found that 48 h of mechanical ventilation with 8 cm H_2_O PEEP application can significantly increase the expression of TGF-β1 accompanied by α-SMA in the diaphragms of healthy rabbits. This study may provide a potential therapeutic target to guide clinical practices preventing PEEP application-induced diaphragm dysfunction.

There were several limitations in this study. First, although fibrosis leads to skeletal muscle weakness, compared to collagen deposition, measurements of muscle contractile properties may better describe diaphragm weakness. Due to the limitation of the experimental conditions, direct measurements of muscle contractile properties were absent. Thus, we carried out SBT test to indirectly reflect the function of diaphragms between the MV group and the MV + PEEP group. However, the negative result of SBT test (as shown in Table 2) didn’t imply that fibrosis has no effect on diaphragmatic function, because the accuracy of SBT test on representing diaphragm function may be not enough to reflect the effect of diaphragm fibrosis on diaphragm function. In fact, the SBT test is not a perfect predictor of respiratory function. In clinical practice, there are also cases where SBT test passed but extubation failed.

## Conclusions

Upon comparing the diaphragm between mechanically ventilated rabbits with/without PEEP application, we demonstrated that PEEP application for 48 h in mechanically ventilated rabbits will cause collagen deposition and fibrosis in the diaphragm. Moreover, activation of the TGF-β1 signaling pathway and myofibroblast differentiation may be the potential mechanism of this diaphragmatic fibrosis. These findings might provide novel therapeutic targets for PEEP application-induced diaphragm dysfunction.

## Supplementary Information

**Additional file 1: Table S1.** Primers used in Quantitative RT-qPCR study. Data are expressed as mean ± SD. **Table S2.** Respiratory monitoring parameters in MV group and MV+PEEP group. Data are expressed as mean ± SD or median (P25, P75). **Table S3.** Patient-ventilator asynchrony in MV group and MV+PEEP group. Data are expressed as mean ± SD or median (P25, P75).

## Data Availability

The datasets used and/or analysed during the current study are available from the corresponding author on reasonable request.

## Abbreviations

PEEP: Positive end-expiratory airway pressure
MV: Mechanical ventilation
RR: Respiratory rate
V_T_: Tidal volume
V_etot_: Total minute ventilation
Ppeak: Airway peak pressure
MP: Mechanical power
VIDD: Ventilation-induced diaphragm dysfunction
ECM: Extracellular matrix
SBT: The spontaneous breathing trial
TGF-β1: Transforming growth factor-β1
α-SMA: Alpha smooth muscle Actin
