# Supplementary material for: PEEP application during mechanical ventilation contributes to fibrosis in the diaphragm
Source: Respir Res. 2023 Feb 13;24:46. doi: 10.1186/s12931-023-02356-y (PMC9926671; doi:10.1186/s12931-023-02356-y)
Supplement: Supplementary file 1 — Additional file 1: Table S1. Primers used in Quantitative RT-qPCR study. Data are expressed as mean ± SD. Table S2. Respiratory monitoring parameters in MV group and MV+PEEP group. Data are expressed as mean ± SD or median (P25, P75). Table S3. Patient-ventilator asynchrony in MV group and MV+PEEP group. Data are expressed as mean ± SD or median (P25, P75). [file 12931_2023_2356_MOESM1_ESM.docx]

Table S1. Primers used in Quantitative RT-qPCR study

Data are expressed as mean ± SD

| Gene | Forward Primer | Reverse Primer |
| --- | --- | --- |
| COL1A1 | ACCACTGCAAGAACAGCGTA | TCGTGGAGGACAGTGTAGGT |
| COL1A2 | TCGATCCCAACCAAGGATGC | CAAACTGGGTGCCACCATTG |
| COL3A1 | AACAATGGTAGTCCTGGCGG | CACCGTTCTTACCGGGTTCA |
| COL5A3 | GCGAGACCTGTCTACATCCG | GCGTCCACGTAGGAGAACTT |
| COL6A1 | GGCATCGAGATCTTCGTGGT | GGTCTGGTAGAACACGCCTC |
| COL6A2 | AGGACCCCAAGTCAGAGACA | GCGATCCACTCGAGGTTCTT |
| COL15A1 | GGAAAAACGGACAAGTCGGC | TCCTCGAATCCAGGTCCCAT |
| COL16A1 | AATGGAGCAGCAGTACCCAC | AGCTTTGGCCACTTATCCCC |
| TGF-β1 | CTGGAACGGGCTCAACATCT | CAGGTCCTTGCGGAAGTCAA |
| FN | GGTTTGGGGTCAATAAGGAAGC | TGTTCCTACAGTGTTGCGGG |
| THBS1 | ACTCGGGGCAGGAAGACTAT | GGATTGTGGTTGTAGGGGCA |
| THBS3 | AAGATCCGGACAGCCCTACT | AATCTTGCCCACGACAGAGG |

Table S2. Respiratory monitoring parameters in MV group and MV+PEEP group.

Data are expressed as mean ± SD or median (P25, P75).

|  | MV | MV+PEEP | *p* |
| --- | --- | --- | --- |
| respiratory cycle | 108115.33 ±2704.50 | 105889.67 ±5973.31 | 0.207 |
| I:E | 0.61(0.58 , 0.84) | 0.61(0.55 , 0.78) | 0.522 |
| RR | 41.16(40.00 , 42.41) | 41.01(39.44 , 41.34) | 0.522 |
| VT(ml) | 19.68(19.47 , 19.93) | 19.80(19.26 , 20.07) | 0.337 |
| Ve tot(L) | 0.83 ± 0.06 | 0.79 ± 0.05 | 0.262 |
| PEEP(cm H_2_O) | 0.25 ± 0.11 | 7.9 ± 0.17*** | ＜0.001 |
| Ppeak(cm H_2_O) | 11.52 ± 1.24 | 15.88 ± 0.85*** | ＜0.001 |
| MP(J/min) | 0.66 ± 0.09 | 0.99 ± 0.05*** | ＜0.001 |

Table S3. Patient-ventilator asynchrony in MV group and MV+PEEP group.

Data are expressed as mean ± SD or median (P25, P75).

|  | MV | MV+PEEP | *p* |
| --- | --- | --- | --- |
| Ineffective Efforts (%) | 0.17 (0.09 , 0.49) | 0.11 (0.08 , 0.33) | 0.699 |
| Double Triggering (%) | 0.20 (0.16 , 0.39) | 0.43 (0.23, 0.59) | 0.180 |
